# The prey’s perspective on the rise of predatory publishing

**DOI:** 10.17179/excli2023-6392

**Published:** 2023-08-23

**Authors:** Leandro Machado Camargo, Mykyta Smirnov, Igor Lima Maldonado

**Affiliations:** 1 Faculdade de Medicina, Universidade Salvador, Salvador, Brazil; 2 UMR 1253, iBrain, Université de Tours, Inserm, Tours, France; 3 SU 'Uzhhorod National University', Uzhhorod, Ukraine

## ⁯

The growth of the internet and electronic communication has led to significant changes in the scientific publishing market. One of these changes is the emergence of open-access journals, which has enabled the rapid and widespread dissemination of scientific material. Unfortunately, there has also been an explosion in the number of predatory journals (Quintans-Júnior et al., 2023[4]).

Some publications have discussed the rise of those journals, including the consequences of the trend and ways to combat it (Davis, 2009[2]; Beall, 2013[1]; Shen and Björk, 2015[5]; Quintans-Júnior et al., 2023[4]). Although these are important issues, one critical yet rarely mentioned aspect is *why* researchers become prey. To address this question, we would like to describe some of the mechanisms through which many colleagues fall victim to this harmful practice.

The rise of predatory journals has been facilitated by an increasing worldwide emphasis on using bibliometric criteria to evaluate academic productivity. Moreover, the growing number of potential authors has notably intensified the competition to publish. For young researchers, career advancement, including their eligibility for academic positions, is now contingent on their performance according to a few bibliometric indicators. Faced with this productivity pressure (the “publish or perish” model), despite limited publication funds, unsuspecting early-career researchers may perceive low-quality open-access (meaning “pay-to-publish”) journals as more viable alternatives to the reputable periodicals where they would compete with more experienced peers from well-established institutions.

Predatory publishing impacts both current and future generations of scientists. A model in which researchers must pay to publish in the early stages of their academic careers represents a shortcoming of career training for scientists, if not a failure. Thus, in addition to being financially manipulative, predatory publishing may influence how researchers behave in relation to scientific communication.

Predatory journals consistently exploit researchers who are not fully proficient in English. Although the predominance of one language in the publishing industry has enabled communication across many disciplines, it has also created an arbitrary standard that exacerbates the opportunity inequality between native and non-native English-speaking authors (Meneghini and Packer, 2007[3]). In certain areas, acquiring adequate English proficiency can require an investment of energy and resources that is neither realistic nor inclusive. Publishers exploit this situation by offering expensive editorial assistance services to non-native English speakers.

Compounding this, many educational and research institutions in underdeveloped countries require their faculty to publish in scientific journals indexed in internationally acclaimed databases, such as Scopus or Web of Science, or in journals published in countries belonging to the Organization for Economic Cooperation and Development (OECD). Ph.D. students are often required to have one such publication before they can defend their thesis. However, in countries that have no local indexed journals in most disciplines and an insufficient level of English proficiency, meeting this requirement can become a crusade. Moreover, studies that are locally important (e.g., public health research) often have limited international appeal. All things considered, early career researchers may avoid submitting their papers to established foreign periodicals, thinking they will be rejected by default. And even if tried, those high-profile, peer-reviewed, open-access journals are often not accessible to poorly funded early-career researchers because of expensive publication fees.

This is the context in which predatory journals swoop in to seize their prey. They lure victims with promises of lower publication fees, faster processing times, and a less demanding peer review process. Some researchers willingly participate in the system, and some are unknowingly scammed, but in either case, the ultimate goal of successful publication in a foreign journal is attained. Shen and Björk evaluated scientific journals produced by 613 predatory publishers on Beall's List between 2010 and 2014 (Beall, 2013[1]; Shen and Björk, 2015[5]). They found that at least three-quarters of the authors in those journals were from Asia or Africa, both of which comprise many low- and middle-income countries.

Overall, a substantial proportion of scientific authors worldwide belong to the demographic targeted by predatory journals: young, early-career scientists who live in low- and middle-income countries and have little experience, limited access to publication grants, and substandard English proficiency. Of course, these vulnerabilities do not apply to everyone, but they do create an abundance of easy targets for exploitation. Predators quickly identify the most susceptible targets. It is, therefore, worth noting that the recent proliferation of predatory journals has been facilitated (and perhaps even accelerated) by the insufficient absorption of the new generation of authors into the publishing market despite the institutional requirement that they publish frequently to ensure their career advancement.

Publishing in predatory journals has severe consequences for both the authors and the scientific community and must be unequivocally avoided. The information we present here should not be taken as any form of justification. Nevertheless, to effectively combat the rise of predatory journals, we must confront reality, comprehend the factors that allow authors to fall into their grasp, and acknowledge that the scientific community, in part, has contributed to the environment that allows these predators to thrive.

## Declaration

### Disclosure

The authors declare that they have no conflict of interest.

### Acknowledgments

None.

### Funding

None.

#### ⁯
